# Serum COMP and Vitamin D as a Biomarker for Articular Cartilage Degeneration in Knee Osteoarthritis: Correlation with USG and MRI Findings

**DOI:** 10.3390/diagnostics16010119

**Published:** 2026-01-01

**Authors:** Radiyati Umi Partan, Agus Mahendra, Murti Putri Utami, Khoirun Mukhsinin Putra, Surya Darma, Muhammad Reagan, Putri Muthia, Afifah Salshabila Radiandina, Hermansyah Hermansyah, Ziske Maritska

**Affiliations:** 1 Division of Rheumatology, Department of Internal Medicine, Dr. Mohammad Hoesin General Hospital, Faculty of Medicine, Sriwijaya University, Palembang 30126, Indonesia; 2 Department of Internal Medicine, Dr. Mohammad Hoesin General Hospital, Faculty of Medicine, Sriwijaya University, Palembang 30126, Indonesia; 3 Department of Medical Biology, Dr. Mohammad Hoesin General Hospital, Faculty of Medicine, Sriwijaya University, Palembang 30126, Indonesia

**Keywords:** knee osteoarthritis, cartilage oligomeric matrix protein, ultrasonography, magnetic resonance imaging

## Abstract

**Background/Objectives**: Osteoarthritis (OA) remains a global health problem, as it can cause permanent joint damage, leading to irreversible disability. Therefore, there is a need for accessible and non-invasive alternative examinations, such as USG, serum COMP, and 25-hydroxyvitamin D [25(OH)D] assessment. This study aims to analyze the correlation between serum COMP and 25(OH)D levels and the degree of articular cartilage degradation in patients with knee OA, based on findings from USG and MRI examinations. **Methods**: A cross-sectional analytical study was conducted at Mohammad Hoesin Hospital, Palembang, from December 2024 to August 2025. 31 patients diagnosed with knee OA based on the 1990 American College of Rheumatology (ACR) classification criteria were enrolled. Serum COMP and 25(OH)D levels were measured. All patients underwent standardized USG and MRI examinations of the knee. Spearman’s rank correlation coefficient was used for statistical analysis. **Results**: The majority of the study subjects were female, comprising 23 (74.2%). The mean age was 63.90 ± 7.77 years with a body mass index of 25.46 ± 5.51 kg/m^2^. Most subjects were engaged in heavy physical activity 17 (54.8%). Laboratory examination showed serum COMP levels with a median of 869 ng/mL and a range of 136–3302 ng/mL. Meanwhile, the 25(OH)D level demonstrated a mean value of 24.84 ± 7.33 ng/mL. The analysis revealed a strong and statistically significant positive correlation between serum COMP levels and the degree of articular cartilage degradation in knee OA. This correlation was observed in both USG (r = 0.61; *p* < 0.001) and MRI assessments (r = 0.72; *p* < 0.001). In contrast, serum 25(OH)D levels showed no significant correlation with cartilage degradation. The correlation coefficient between 25(OH)D levels and USG-assessed cartilage degradation was r = −0.12 (*p* = 0.51), and for MRI assessment, it was r = 0.17 (*p* = 0.92). **Conclusions**: A strong and significant positive correlation exists between serum COMP levels and the degree of articular cartilage degradation based on USG (r = 0.61; *p* < 0.001) and MRI (r = 0.72; *p* < 0.001). In contrast, serum 25(OH)D levels showed no significant correlation with cartilage degradation, implying that 25(OH)D may not directly reflect the extent of structural cartilage damage in knee osteoarthritis. This finding proves that an increase in serum COMP levels is associated with an increase in the degree of articular cartilage degradation in knee OA as measured by both USG and MRI.

## 1. Introduction

Indonesia is the largest archipelagic country in the world, consisting of more than 18,000 islands stretching from Sabang to Merauke. Its vast and dispersed geographical condition poses a unique challenge in achieving equitable healthcare services, particularly in the availability of modern diagnostic facilities. One of the diagnostic tools with high diagnostic value, such as MRI, remains limited and unevenly distributed across various regions in Indonesia [1]. This infrastructural limitation leads to delayed diagnosis and evaluation of degenerative musculoskeletal diseases such as knee OA, whose prevalence continues to increase along with the rising life expectancy of the population [1,2]. Osteoarthritis is a degenerative joint disease characterized by progressive damage to the articular cartilage, changes in the subchondral bone, and synovial inflammation. In advanced stages, OA can lead to joint deformity and permanent movement limitation [3]. Many elderly patients experience prolonged immobilization or long-term bed rest due to pain and loss of joint function. Prolonged immobility not only reduces quality of life but also increases the risk of systemic complications such as muscle mass loss (sarcopenia), metabolic disturbances, and a higher incidence of cerebrocardiovascular diseases such as stroke and ischemic heart disease [4]. Magnetic resonance imaging (MRI) is currently considered the gold standard for detecting articular cartilage damage since it provides comprehensive imaging of soft tissues [5]. However, MRI use in Indonesia remains limited due to high cost, long examination times, and uneven availability, especially in remote areas. This situation highlights the necessity for new diagnostic methods that are more affordable, accessible, and still provide good diagnostic value for early detection and monitoring of OA progression [4,5]. One of the promising imaging modalities is ultrasonography (USG) combined with serum COMP and 25-hydroxyvitamin D [25(OH)D] level measurement. With technological advancements, USG can now assess cartilage degradation and integrity, detect joint effusion, osteophytes, and periarticular tissue changes associated with OA. Ultrasonography is non-invasive, radiation free, relatively low cost, and can be performed repeatedly on various anatomical areas in a short period of time. Therefore, ultrasound may serve as a potential real-time morphological assessment tool for joint damage [6,7,8]. In addition to imaging modalities, recent studies have highlighted the role of biochemical biomarkers in detecting and monitoring cartilage tissue damage. One of the most widely studied biomarkers is Cartilage Oligomeric Matrix Protein (COMP), a non-collagen glycoprotein abundant in the extracellular matrix of cartilage. Elevated serum COMP levels reflect increased cartilage degradation and matrix remodeling activity, thus representing disease severity and progression. Several previous studies, such as those by Clark et al. (1999) and Verma & Dalal (2013), have demonstrated a significant positive correlation between serum COMP levels and both OA severity and joint pain [9,10]. Similarly, Kambayana et al. (2014) reported a correlation between serum COMP levels and radiographic grading of knee OA [11]. In the Framingham study, subjects with the lowest serum 25-hydroxyvitamin D [25(OH)D] levels (<27 ng/mL) and those in the borderline range (27.9–33.0 ng/mL) had a threefold increased risk of developing progressive knee osteoarthritis [12]. Partan & Putra (2024) discovered a significant and strong negative correlation between 25-hydroxyvitamin D [25(OH)D] and COMP serum in knee osteoarthritis [13]. Considering Indonesia’s vast geography and the limited availability of MRI facilities in many regions, alongside the increasing need for efficient and affordable diagnostic methods, there is a growing demand for non-invasive, accessible, and effective approaches for early detection and evaluation of OA progression. USG, serum COMP and 25-hydroxyvitamin D [25(OH)D] measurement have the potential to serve as complementary tools, providing both morphological and biochemical insights into joint damage for a more comprehensive assessment of articular cartilage degradation. Therefore, this study aims to analyze the correlation between serum COMP and 25-hydroxyvitamin D [25(OH)D] levels to the degree of articular cartilage degradation in patients with knee OA using USG and MRI examinations at Mohammad Hoesin Hospital, Palembang.

## 2. Materials and Methods

This study is an analytical observational research with a cross-sectional design, and it fulfilled the specified inclusion criteria. The research was performed at the Rheumatology Division of Dr. Mohammad Hoesin General Hospital in Palembang from December 2024 to August 2025. The study was conducted in accordance with the guidelines outlined in the Declaration of Helsinki by the researchers. This study received ethical approval from the Health Research Ethics Committee of Mohammad Hoesin General Hospital, Palembang, under approval number DP.04.03/D.XVIII.06.08/ETIK/284/2024, date 17 December 2024.

### 2.1. Study Population

The study population consisted of all patients from the accessible population who met the predefined inclusion and exclusion criteria. The study enrolled purposive participants who were eligible for inclusion if they were diagnosed with knee osteoarthritis (OA) according to ACR 1990 [13] classification criteria, were willing to participate voluntarily, and provided written informed consent prior to enrollment. Exclusion criteria included patients who declined participation, those with autoimmune diseases that could potentially affect serum biomarker levels, and individuals with knee OA who were unable to flex the knee joint, which would hinder optimal USG and MRI assessments.

### 2.2. Participant Selection

**Figure 1:**
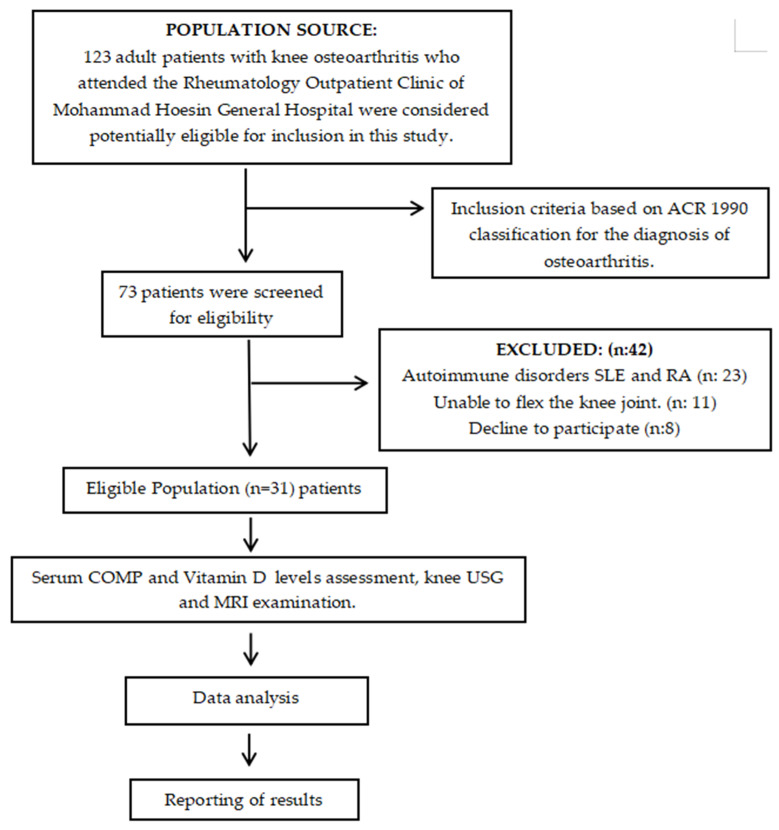
Patients’ recruitment protocol.

A total of 123 adult patients with clinically diagnosed knee osteoarthritis who attended the Rheumatology Outpatient Clinic of Mohammad Hoesin General Hospital, Palembang, were initially screened for potential eligibility. Among these, 73 patients met the inclusion criteria based on the American College of Rheumatology (ACR) 1990 classification criteria for osteoarthritis. Subsequently, 42 patients were excluded according to the predefined exclusion criteria. The reasons for exclusion included the presence of autoimmune disorders such as systemic lupus erythematosus (SLE) or rheumatoid arthritis (RA) in 23 patients, inability to flex the knee joint which hindered optimal ultrasonographic and MRI assessment in 11 patients, and refusal to participate in 8 patients. After applying all inclusion and exclusion criteria, a total of 31 eligible patients were enrolled in the study. All participants underwent serum COMP and 25(OH)D levels measurement, knee USG and MRI examinations, as seen in Figure 1. Ultrasonography (USG) and magnetic resonance imaging (MRI) examinations were independently performed by professional specialists—a rheumatology consultant and a radiologist. Each examiner was blinded to the clinical data and the results of the other assessments, and there was no interaction between them to avoid examination bias. The collected data were subsequently analyzed and the results were reported according to the research protocol. The assessment focused on two primary variables. The independent variable was the serum COMP concentration the BioVendor TRD 194080200 Human Cartilage Oligomeric Matrix Protein (COMP) ELISA reagent, with results expressed in ng/mL (BioVendor—Laboratorní medicína a.s., Brno, Czech Republic). Catalog No.: RD194080200, Lot: E24-022, Expiration Date: 30 May 2026. The standard calibration range was 4–128 ng/mL, with a limit of detection of 0.4 ng/mL. In contrast, the serum 25(OH)D concentration was assessed via biochemical blood testing. The dependent variable was the degree of articular cartilage degradation in knee osteoarthritis, which was evaluated based on findings from knee USG and MRI examinations. In Ultrasound assessment, patients were examined in the supine position with the knee in slight flexion of approximately 30° to facilitate visualization of the articular cartilage surface. The femoral articular cartilage was evaluated using an anterior suprapatellar approach, with the transducer placed longitudinally along the midline of the knee above the patella and slowly moved distally until the medial and lateral femoral condyles were visualized. Each knee was examined systematically in the medial and lateral compartments, and the imaging findings were documented as digital images. The degree of cartilage degradation obtained from the ultrasound examination was then recorded for correlation analysis. In MRI assessment, subjects were placed in the supine position, with the examined knee in full extension and immobilized to minimize motion artifacts. The imaging protocol included standard sequences for cartilage evaluation, namely T1-weighted sagittal and coronal images to assess general anatomy and joint contours; proton density (PD) sequences with fat suppression in the sagittal, coronal, and axial planes to evaluate cartilage thickness, surface integrity, and the presence of subchondral sclerosis. The articular cartilage of the medial and lateral femoral condyles and the patella was evaluated systematically. The degree of cartilage degradation obtained from the MRI examination was then recorded for correlation analysis. The severity of cartilage damage was graded using the International Cartilage Repair Society (ICRS) grading system for USG and MRI evaluation. The severity of cartilage damage was graded using the International Cartilage Repair Society (ICRS) grading system for USG and MRI evaluation [14]. In addition, supporting data—including patients’ age, sex, occupation, educational level, nutritional status, and physical activity level—were recorded as complementary variables for further analysis.

### 2.3. Statistical Analyses

Data analysis was conducted in several sequential stages. Categorical variables were presented in tables and descriptive narratives, including frequencies and percentages. Numerical data were analyzed using the same testl sequential stages. The normality of data distribution was assessed using the Shapiro–Wilk to determine data distribution. Variables with normal distribution (*p* > 0.05) were presented as mean ± standard deviation (SD), while non-normally distributed data (*p* < 0.05) were expressed as median and range (minimum–maximum). All research results were described narratively and supplemented with frequency distribution tables and graphical statistical representations. We used Spearman’s rank correlation test on serum COMP levels and the Pearson’s rank correlation test on 25(OH)D to find out how the degree of articular cartilage deterioration measured by USG and MRI was related. A *p*-value < 0.05 was considered statistically significant. The strength of correlation was interpreted according to the correlation coefficient (r) range from −1 to +1, where values closer to ±1 indicated stronger correlation, and values near 0 indicated weak or no correlation. All statistical analyses were performed using IBM SPSS Statistics version 25.0 (IBM Corp., Armonk, NY, USA) with a significance level set at α = 0.05.

## 3. Results

### 3.1. Baseline Characteristic

Table 1 shows the general characteristics of the research subjects, including age, gender, nutritional status, and physical activity. In the statistical tests performed, all research subjects were OA patients with a mean age of 63.90 ± 7.765 years. The most common age groups were the 51–60 years range (38.7%) and the 61–70 years range (38.7%). Gender was dominated by 23 females (74.2%) compared to 8 males (25.8%). The majority of patients had an overweight (35.5%) and obese (29%) nutritional status, totaling 64.5%, with a mean Body Mass Index (BMI) of 26.05 ± 5.369. The physical activity level most frequently observed was heavy physical activity (54.8%).

### 3.2. Characteristics of Articular Cartilage Degradation Degree Based on USG

**Figure 2:**
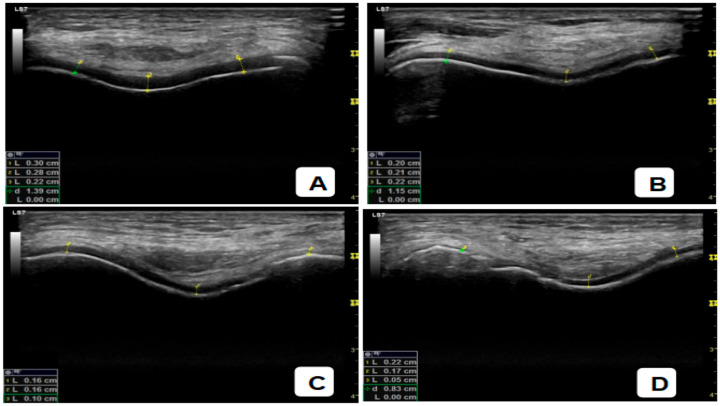
Grades of articular cartilage degradation in knee osteoarthritis-based USG (ICRS Score): (**A**). Grade 1; (**B**). Grade 2; (**C**). Grade 3; (**D**). Grade 4.

The degree of articular cartilage degradation in knee osteoarthritis based on Ultrasound (USG) using the ICRS classification. Grade 1 (Figure 2A) shows only mild superficial changes, while Grade 2 (Figure 2B) indicates thinning of <50% of the cartilage thickness. Grade 3 (Figure 2C) is characterized by damage to >50% of the thickness, and Grade 4 (Figure 2D) demonstrates complete cartilage loss with exposed subchondral bone. This sequence of images clearly visualizes the progression of cartilage damage from mild-to-severe stages.

Table 2 presents the general characteristics of articular cartilage degradation degree based on knee USG measurements. According to the ICRS classification using USG, the majority of research subjects showed a higher degree of articular cartilage degradation. The distribution is 6.5% at Grade 1, 25.8% at Grade 2, 32.3% at Grade 3, and 35.5% at Grade 4. This illustrates that most of the patients examined were already in the stage of moderate to severe damage.

### 3.3. Characteristics of Articular Cartilage Degradation Degree Based on MRI

**Figure 3:**
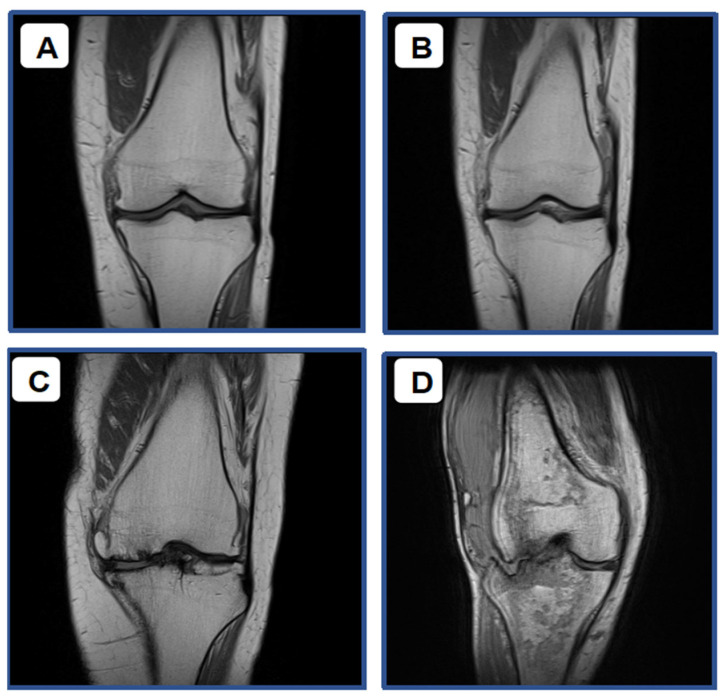
Degree of Articular Cartilage Degradation in Knee OA based on MRI (Outerbridge Score): (**A**). Grade 1; (**B**). Grade 2; (**C**). Grade 3; (**D**). Grade 4.

Figure 3 provides a clear visualization of the progressive degrees of articular cartilage degradation in knee osteoarthritis, as assessed by MRI using the Outerbridge classification. The progression begins with Grade 1 (Figure 3A), where the damage is limited to mild superficial changes. This stage is characterized by an increased signal intensity visible only within the surface layer of the cartilage, while the deeper structure remains relatively intact. Moving to Grade 2 (Figure 3B), the damage involves definite cartilage thinning, with the lesion penetrating less than 50% of the total cartilage thickness, and the surface contour starts to become noticeably irregular. Grade 3 (Figure 3C) marks a more severe stage, showing a deeper lesion that involves more than 50% of the cartilage thickness. At this point, the defect is wide and extensive, extending close to the subchondral bone. Finally, Grade 4 (Figure 3D) represents the most advanced stage of damage. This image shows near-total cartilage loss, with clear exposure of the subchondral bone, signifying severe and late-stage deterioration of the joint surface.

Table 3 displays the general characteristics of the degree of articular cartilage degradation as measured using knee MRI. Based on the Outerbridge classification using MRI, the distribution pattern is relatively similar to that found by USG examination, though with slightly different proportions. A total of 12.9% of subjects were at Grade 1, 35.5% at Grade 2, 22.6% at Grade 3, and 29% at Grade 4. Thus, MRI also detected a greater number of cases in the moderate-to-advanced stages. Overall, both USG and MRI demonstrated that the majority of patients exhibited articular cartilage degradation at Grades 2–4, with distribution peaks at Grades 3 and 4 for USG, and Grades 2 and 4 for MRI. This confirms that, in this study population, most patients presented with fairly significant cartilage damage.

### 3.4. Characteristics of Serum COMP and 25(OH)D Analysis

The laboratory characteristics of the research subjects based on serum COMP levels were examined. The median COMP level obtained was 869 ng/mL, with a value range spanning from 136 to 3302 ng/mL. Meanwhile, the serum 25(OH)D concentration showed a mean value of 24.84 ng/mL with a standard deviation (SD) of ±7.33 ng/mL.

### 3.5. Determination of Cut-Off Points for Serum COMP Concentrations Based on Knee USG and MRI Findings

**Figure 4:**
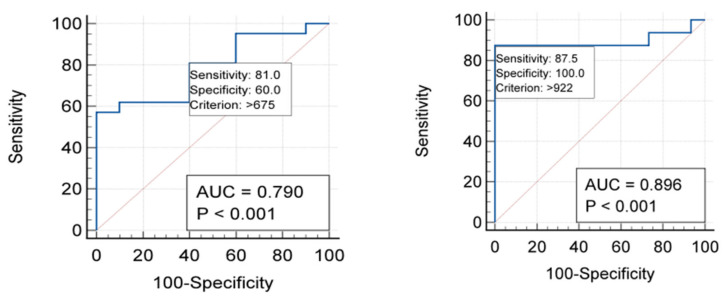
Serum COMP Cut-off Thresholds Determined by Knee USG and MRI.

Serum COMP levels are measured as a continuous variable, necessitating the determination of a cut-off point (threshold value) for accurate diagnostic interpretation to differentiate the individuals being measured. For this purpose, Receiver Operating Characteristic (ROC) curve analysis was used to evaluate the discriminative ability of the serum COMP levels. In this study (Figure 4), the calculation of the cut-off points for serum COMP levels based on the degree of articular cartilage degradation in knee OA using USG yielded a threshold value of >675 ng/mL, with a sensitivity of 81% and a specificity of 60%. Conversely, the cut-off points for serum COMP levels based on the degree of articular cartilage degradation in knee OA using MRI yielded a threshold value of >922 ng/mL, with a sensitivity of 87.5% and a specificity of 100%.

Table 4 provides the relationship between serum COMP levels and the degree of articular cartilage degradation based on knee USG examination in OA patients. Out of the total 31 subjects, 21 individuals had serum COMP levels of ≥675 ng/mL. The majority of this group (17 subjects) were classified into Grade 3 and 4 degradation, while only 4 subjects were in Grade 1 and 2. Conversely, among subjects with serum COMP levels of <675 ng/mL, the majority (6 out of 10 subjects) fell into Grade 1 and 2, with only 4 subjects classified as Grade 3 and 4. Statistical analysis using the Chi-Square test showed a *p*-value of 0.040 (*p* < 0.05), which indicates a statistically significant relationship between serum COMP levels and the degree of articular cartilage degradation based on knee USG examination.

Table 5 illustrates the relationship between serum COMP levels and the degree of articular cartilage degradation based on MRI examination. Out of the 31 subjects, 17 individuals had serum COMP levels of ≥922 ng/mL. Almost all of this group (14 subjects) fell into the Grade 3 and 4 degradation categories, while only 3 subjects were in Grade 1 and 2. Conversely, in the group with serum COMP levels of ≤922 ng/mL, the vast majority (15 out of subjects) were classified as Grade 1–2, with only 2 subjects falling into Grade 3–4. The statistical analysis using the Chi-Square test showed a *p*-value of <0.0001 (*p* < 0.05), which confirms a significant relationship between serum COMP levels and the degree of articular cartilage degradation based on knee MRI examination.

### 3.6. Analysis of Serum 25(OH)D Levels to the Articular Cartilage Degradation Based on USG and MRI in Knee Osteoarthritis

Table 6 presents the laboratory characteristics of the study subjects based on serum 25(OH)D levels. According to the Institute of Medicine classification, 74.2% of the subjects had low 25(OH)D levels, while 25.8% had normal levels. This finding indicates that, in the study population, the majority of patients presented with a significantly reduced serum 25(OH)D concentration.

Table 7 illustrates the relationship between serum 25(OH)D levels and the degree of articular cartilage degradation in patients with knee osteoarthritis (OA) as assessed by ultrasound (USG). Out of 31 total subjects, 23 individuals (74.2%) had low serum 25(OH)D levels (deficient or insufficient), while 8 individuals (25.8%) had normal levels. Among those with low vitamin D, 15 participants (65%) were classified as having severe cartilage degradation (Grade 3–4), and 8 participants (35%) were in the mild-to-moderate group (Grade 1–2). In contrast, among those with normal 25(OH)D levels, 4 participants (50%) were in the mild-to-moderate group (Grade 1–2), and 4 participants (50%) were in the severe group (Grade 3–4). Fisher’s exact test yielded a *p*-value of 0.040 (*p* < 0.05), indicating a statistically significant association between serum 25(OH)D levels and the degree of articular cartilage degradation in knee OA based on USG findings. These results suggest that lower serum 25(OH)D levels are associated with greater severity of cartilage degradation in patients with knee osteoarthritis.

Table 8 illustrates the relationship between serum 25(OH)D levels and the degree of articular cartilage degradation based on MRI findings. Among the 31 subjects, 8 individuals had normal serum 25(OH)D levels, with half of them (4 subjects) classified into the severe group (cartilage degradation Grades 3–4), while the remaining four subjects belonged to the mild-to-moderate group (Grades 1–2). Conversely, in the group with low serum 25(OH)D levels (deficient and insufficient) comprising 23 subjects, 11 subjects (47.8%) were categorized as mild-to-moderate (Grades 1–2), whereas 12 subjects (52.2%) were classified as severe (Grades 3–4) based on knee MRI evaluation. Statistical analysis using Fisher’s Exact Test yielded a *p*-value of 0.928 (*p* > 0.05), indicating no significant relationship between serum 25(OH)D levels and the degree of articular cartilage degradation in patients with knee osteoarthritis.

### 3.7. Correlation Between Serum COMP and 25(OH)D Levels and Articular Cartilage Degradation Grade in Knee Osteoarthritis (OA) Patients Using USG and MRI

The analysis revealed a statistically significant positive correlation between serum COMP levels and the degree of articular cartilage degradation in knee osteoarthritis (OA), as assessed by both USG (r = 0.61; *p* < 0.001) and MRI (r = 0.72; *p* < 0.001), indicating a strong correlation strength, as seen Table 9. These findings demonstrate that increased serum COMP levels are associated with higher degrees of articular cartilage degradation in knee OA, as detected by both USG and MRI evaluations. In contrast, serum 25(OH)D levels showed no significant correlation with cartilage degradation. The correlation coefficient between 25(OH)D levels and USG assessed cartilage degradation was r = −0.12 (*p* = 0.51), and for MRI assessment, it was r = 0.17 (*p* = 0.92). These weak and statistically insignificant correlations suggest that serum 25(OH)D levels do not have a measurable relationship with the degree of articular cartilage damage in knee OA based on either USG or MRI evaluations. Overall, the results highlight that serum COMP serves as a more reliable biomarker for reflecting cartilage degeneration severity in knee osteoarthritis compared to 25(OH)D levels.

**Table 9:** Correlation of Serum COMP and 25(OH)D Levels with Articular Cartilage Degradation Grade in Knee OA (USG and MRI).

Correlation	r	*p*-Value
Serum COMP Levels vs. Degree of Articular Cartilage Degradation in Knee OA Assessed by USG	0.61	<0.001
Serum COMP Levels vs. Degree of Articular Cartilage Degradation in Knee OA Assessed by MRI	0.72	<0.001
Serum 25(OH)D Levels vs. Degree of Articular Cartilage Degradation in Knee OA Assessed by USG	−0.12	0.51
Serum 25(OH)D Levels vs. Degree of Articular Cartilage Degradation in Knee OA Assessed by MRI	0.17	0.92

## 4. Discussion

In this study, the mean age was found to be 63.90 ± 7.765 years, with the highest proportion of subjects belonging to the 51–60 years age group (38.7%) and the 61–70 years age group (38.7%). These figures indicate that the incidence of OA is directly proportional to increasing age in the population, particularly among the elderly. Osteoarthritis is most prevalent in the elderly because the aging process causes cartilage degradation, chondrocytes lose their regenerative capacity, the quality of synovial fluid and the elasticity of supporting joint tissues decline, and the accumulation of mechanical load and microtrauma throughout life ultimately accelerate joint damage [15]. In this study, there were 31 subjects consisting of 23 females (74.2%) and 8 males (25.8%). This aligns with the theory stating that the prevalence of knee OA is higher in women than in men. Several pieces of the literature suggest that women are more susceptible to OA due to the decline in estrogen post-menopause, which plays a role in maintaining cartilage metabolism. Other important factors contributing to the higher prevalence of OA in women include anatomical structural differences and variations in knee joint kinematics, which place a greater load-bearing stress on women compared to men [16,17]. The nutritional status of the research subjects, measured by Body Mass Index (BMI), was dominated by the overweight and obese groups, totaling 51.6%. This figure suggests that the incidence of OA is directly proportional to nutritional status, supporting the hypothesis that individuals who are overweight or obese have a higher risk factor for developing OA. A prospective meta-analysis published by Zheng H et al. found that overweight individuals have an approximately 2.5-fold higher risk, and obese individuals up to a 4.6-fold higher risk, of experiencing knee OA compared to those with normal weight. The risk also increases by approximately 35% for every 5 kg/m^2^ increase in BMI [18]. Obesity can increase the mechanical load (biomechanics) on weight-bearing joints, which in turn increases the production of pro-inflammatory cytokines, mediating the catabolic process of OA. This mechanical stress on the weight-bearing joints activates chondrocytes and accelerates the progressive degradation of the cartilage [19,20]. In this study, the majority of subjects reported heavy physical activity (54.8%), while a smaller portion reported light activity (32.3%) and moderate activity (12.9%). This is likely influenced by the characteristics of the subjects, most of whom are elderly individuals whose daily routines still involve physical labor, such as intensive household activities, gardening, or other productive work requiring significant effort. Furthermore, within the cultural and social context of Indonesian society, many elderly individuals, particularly women, remain active in domestic duties, caring for grandchildren, shopping at traditional markets, walking, gardening, and engaging in informal economic activities that are physically demanding. This necessity is often greater among those from lower-middle socio-economic backgrounds, who must work harder to meet their living needs. This contrasts with populations in developed countries, where the elderly tends to be more sedentary. The elderly in this setting still rely on direct physical activity such as walking, cycling, and manually carrying goods rather than using modern vehicles or technology, resulting in a high level of physical activity classified as heavy [20]. In this study, we measured serum COMP levels, followed by examination using the ELISA protein PCR technique in an accredited laboratory. The results showed a Median (Min–Max) of 869 (136–3302) ng/mL, with a statistically significant *p*-value (*p* = 0.005). This finding interprets that the COMP level functions as a strong predictor variable and has a significant influence. Akinmade A et al. (2021) [21] demonstrates that sCOMP is significantly higher in patients with knee OA than in those without the disease. Values of sCOMP were also found to increase with severity of knee OA, indicating the possibility of its use as a marker of diagnosis and severity [21]. The potential of COMP as an OA biomarker is currently widely researched. This is because during inflammation, particularly in the cartilage, COMP fragmentation occurs due to the destruction of the extracellular matrix in OA. This COMP is subsequently released into the synovial fluid and then into the blood, meaning that a finding of increased COMP concentration in the serum can indicate ongoing cartilage damage in OA cases. This is supported by a meta-analysis conducted by Zhang J et al. (2018) [22], which showed a significant increase in serum COMP in knee OA participants compared to normal subjects. The use of COMP as an OA biomarker may serve as a marker for the early stages of OA, as pathological signs are often not visible on standard diagnostic radiological examinations during these initial stages. This allows for early intervention and prevention [22]. A study conducted by [23] reported that an increase in COMP levels could be detected 2 weeks after ligament damage and increased gradually until the 12th week, with no peak detected. COMP testing can also be utilized to evaluate treatment effectiveness in OA cases [23]. Feng L. et al. (2021) [24] reported that a strong positive correlation was observed between serum COMP levels and the radiological severity of knee OA (r = 0.62, *p* < 0.001). Similarly, synovial fluid COMP levels also showed a significant correlation with OA severity (r = 0.68, *p* < 0.001) [23]. These findings suggest that COMP, in both serum and synovial fluid, may serve as a potential biomarker reflecting the degree of cartilage degradation and the radiological severity of knee osteoarthritis. The distribution of articular cartilage degradation based on USG findings was as follows: 6.5% at Grade 1, 25.8% at Grade 2, 32.3% at Grade 3, and 35.5% at Grade 4. This illustrates that the majority of patients examined were already in the stage of moderate to severe damage. Similarly, the distribution of articular cartilage degradation in knee osteoarthritis based on MRI findings was as follows: 12.9% of subjects at Grade 1, 35.5% at Grade 2, 22.6% at Grade 3, and 29% at Grade 4. Thus, MRI also detected a greater number of cases in the intermediate to advanced stages. Overall, both USG and MRI demonstrated that the majority of patients exhibited articular cartilage degradation at Grades 2–4, with the distribution peaking at Grades 3 and 4 for USG, and Grades 2 and 4 for MRI. This confirms that, in this research population, most patients presented with significantly damaged cartilage. In terms of assessing synovial abnormalities, several studies suggest that ultrasonography shows very good agreement, though not quite as high as MRI, with a concordance rate of 75% for synovial hypertrophy assessment and 100% for joint effusion assessment [25]. According to Saarakkala et al. (2012) [26], ultrasonography demonstrated a good-to-moderate correlation with arthroscopic evaluation in detecting degenerative changes in the articular cartilage, particularly within the sulcus and medial femoral condyle regions. In the sulcus area, the correlation was relatively strong (*r* = 0.593, *p* < 0.001), whereas in the medial condyle, the correlation was moderate yet remained statistically significant (*r* = 0.465, *p* = 0.003) [26]. These results indicate that USG can serve as a reliable imaging modality for assessing the severity of cartilage degeneration in specific knee regions, although its diagnostic accuracy may vary depending on the anatomical site evaluated. MRI is generally considered the best non-invasive method for assessing joint cartilage due to its high soft tissue contrast. With conventional techniques, MRI can provide detailed information regarding chondral thickness, morphological abnormalities of the chondral surface, signal changes within the cartilage substance, and subchondral bone abnormalities. However, USG is not far behind in depicting the degree of articular cartilage degradation, demonstrating a concordance value that is almost comparable to MRI.

The core finding of this study is the statistically significant positive correlation between serum COMP levels and the degree of articular cartilage degradation in knee OA patients. This relationship proved strong for both imaging modalities used: r = 0.61 based on USG and a slightly stronger r = 0.72 based on MRI (both with *p* < 0.001). These findings unequivocally conclude that an increase in serum COMP levels is directly associated with more severe cartilage degradation as measured by both USG and MRI. To determine the diagnostic usefulness of COMP, ROC curve analysis was performed. The analysis yielded different optimal cut-off points depending on the imaging reference. For degradation assessed by USG, the threshold was determined to be >675 ng/mL, offering a sensitivity of 81% and a specificity of 60%. Conversely, when degradation was assessed by MRI, the optimal threshold was >922 ng/mL, showing a higher sensitivity of 87.5% and a perfect specificity of 100%. Furthermore, Chi-Square tests confirmed a significant relationship between COMP levels and degradation for both USG (*p* = 0.040) and MRI (*p* < 0.0001). This comparison reveals that MRI possesses a significantly higher specificity than USG in assessing the degree of cartilage degradation. In practical terms, this means MRI is superior for confirming the diagnosis (rule-in), while USG, with its relatively high sensitivity, remains a good option for initial detection or screening. This conclusion aligns with the existing literature emphasizing the utility of COMP. Research by Nishida Y et al. (2021) [27] showed that serum COMP levels correlate positively with various risk factors and advanced imaging findings (MRI T2 relaxation time, arthroscopy) even in ACL-deficient patients whose standard radiographs appear normal. This highlights COMP’s potential as an early biomarker capable of detecting subclinical cartilage damage before it is visible on conventional X-rays, thus allowing for earlier intervention and prevention. Supporting the reliability of imaging, other studies confirm that USG holds its own [27]. Brom M et al. (2020) [28] further demonstrated USG’s strong diagnostic performance: the absence of both osteophytes and cartilage degradation on USG has a high negative predictive value (NPV of 92%), making it excellent for ruling out OA. Conversely, the combined presence of both findings yields a high specificity (94%), confirming the diagnosis. Most validity studies show good agreement between USG findings (especially for synovitis) and gold-standard MRI or arthroscopy [28]. Ultimately, the results of this study showing a strong and significant correlation between serum COMP and degradation assessed by both MRI and USG underscore the value of COMP as a diagnostic and prognostic marker for knee OA, capable of identifying patients at risk for disease progression, consistent with other research showing high COMP concentrations persisting over time. While MRI offers superior specificity, USG provides a viable and strongly correlated alternative for clinical assessment. Simultaneously, serum 25(OH)D levels did not exert a substantial influence on cartilage degradation. The correlation between 25(OH)D levels and cartilage deterioration, measured by USG, was r = −0.12 (*p* = 0.51), and by MRI, it was r = 0.17 (*p* = 0.92). These weak and statistically insignificant correlations suggest that serum 25(OH)D levels do not demonstrate a meaningful relationship with the extent of articular cartilage destruction in knee osteoarthritis, evaluated using USG or MRI. The strength of articular cartilage depends on its thickness, volume, and surface defects. These factors include of mechanical loads, synovial inflammation, subchondral bone remodeling, genetic predisposition, and prior joint injury. 25(OH)D metabolism is not the only factor that affects this process [29]. Interventional studies have demonstrated that increasing serum 25(OH)D levels does not alter the progression of pre-existing cartilage degradation. Additionally, meta-analyses and systematic reviews indicate that the association between blood 25(OH)D levels and cartilage degradation is not uniform across all studies [30]. 25(OH)D should not be regarded as a reliable singular biomarker for assessing the condition of articular cartilage in osteoarthritis, as cartilage degeneration is complex and arises from various factors.

This study has many limitations. The study was conducted at a single research institution and was restricted to individuals diagnosed with knee osteoarthritis, as per the ACR 1990 diagnostic criteria. This study employed a cross-sectional design, which only describes the correlation between serum COMP and 25(OH)D levels and the degree of articular cartilage degradation at a single point in time. Therefore, this design cannot evaluate causal relationships or longitudinal changes in COMP and 25(OH)D levels. The findings did not extend to other osteoarthritis groups exhibiting diverse levels of radiographic severity. It is also crucial to note that the study’s sample size was not very large, and we recommend that future studies have a larger sample size to corroborate the results. There is also a chance of selection bias in how study participants were chosen. The non-random sampling technique may compromise the sample’s representativeness and hence impact the study’s outcomes. Lastly, we did not directly evaluate cartilage using high-resolution magnetic resonance imaging or histological examination, which might have provided more objective evidence for efficacy assessment.

## 5. Conclusions

This study demonstrated a strong and statistically significant positive correlation between serum Cartilage Oligomeric Matrix Protein (COMP) levels and the degree of articular cartilage degradation in patients with knee osteoarthritis, as assessed by both USG and MRI. Meanwhile, serum 25(OH)D levels showed no significant correlation with cartilage degradation, implying that 25(OH)D may not directly reflect the extent of structural cartilage damage in knee osteoarthritis. Therefore, serum COMP measurement may be considered a useful non-invasive biomarker for the early evaluation of knee osteoarthritis, facilitating timely detection and management of degenerative joint changes.

## Figures and Tables

**Table 1:** Baseline characteristics.

Variable	Subjects (*n*)	(%)	Mean ± SD	Median (Min–Max)	*p*
Age (years) 51–60 years 61–70 years 71–80 years >80 years			63.90 ± 7.765		0.236
	12 12 7	38.7 38.7 22.6			
Total	31	100			
Gender					
Female	23	74.2			
Male	8	25.8			
Total	31	100			
Body Mass Index			26.05 ± 5.369		<0.001
Severe Underweight (<17)					
Underweight (17–18.4)	1	3.2			
Normal (18.5–25)	10	32.3			
Overweight (25.1–27)	9	29.0			
Obese (>27)	11	35.5			
Total	31	100			
Physical Activity					<0.001
Mild	10	32.3			
Moderate	4	12.9			
Severe	17	54.8			
Total	31	100			

**Table 2:** Articular Cartilage Degradation Degree Characteristics based on USG.

Variable	Subjects (*n*)	(%)
Degree of Articular Cartilage Degradation in Knee OA (ICRS Classification USG)		
Grade 1	2	6.5
Grade 2	8	25.8
Grade 3	10	32.3
Grade 4	11	35.5
Total	31	100

**Table 3:** Articular Cartilage Degradation Degree Characteristics Based on MRI.

Variable	Subjects (*n*)	(%)
Degree of Articular Cartilage Degradation in Knee OA (Outerbridge Classification MRI)		
Grade 1	4	12.9
Grade 2	11	35.5
Grade 3	7	22.6
Grade 4	9	29.0
Total	31	100

**Table 4:** Table of the Relationship Between Serum COMP Levels based on USG in Knee OA.

Serum COMP Level	Articular Cartilage Degradation Degree in Knee OA (USG)		Total (*n*)	*p*-Value
	Grade 3, 4 (*n*)	Grade 1, 2 (*n*)		
>675	17	4	21	0.040
≤675	4	6	10	
Total	21	10	31	

Chi Square-test.

**Table 5:** Table of the Relationship Between Serum COMP Levels based on MRI in Knee OA.

Serum COMP Level	Articular Cartilage Degradation Degree in Knee OA (USG)		Total (*n*)	*p*-Value
	Grade 3, 4 (*n*)	Grade 1, 2 (*n*)		
>922	14	0	17	<0.0001
≤922	2	15	14	
Total	15	16	31	

Chi-Square test.

**Table 6:** Laboratory Characteristics of Study Subjects.

Classification of Serum 25(OH)D Levels	Subjects (*n*)	(%)
Low/Deficient and Insufficient (<10–29 nmol/L)	23	74.2
Normal (>30 nmol/L)	8	25.8
Total	31	100

**Table 7:** Table of the Relationship between Serum 25(OH)D Levels and Cartilage Degradation Degree on USG in Knee OA.

Serum 25(OH)D Level	Articular Cartilage Degradation Degree in Knee OA (USG)		Total (*n*)	*p*-Value
	Grade 3, 4 (*n*)	Grade 1, 2 (*n*)		
Low (Deficient, Insufficient)	15	8	23	0.040
Normal	4	4	8	
Total	19	12	31	

Fisher’s exact test.

**Table 8:** Table of the Relationship between Serum 25(OH)D Levels based on MRI in Knee OA.

Serum 25(OH)D Level	Articular Cartilage Degradation Degree in Knee OA (MRI)		Total (*n*)	*p*-Value
	Grade 3, 4 (*n*)	Grade 1, 2 (*n*)		
Low (Deficient, Insufficient)	12	11	23	0.928
Normal	4	4	8	
Total	16	15	31	

Fisher’s exact test.

## Data Availability

The original contributions presented in this study are included in the article. Further inquiries can be directed to the corresponding author.
